# Platelet-Rich Fibrin in Single and Multiple Coronally Advanced Flap for Type 1 Recession: An Updated Systematic Review and Meta-Analysis

**DOI:** 10.3390/medicina57020144

**Published:** 2021-02-05

**Authors:** Leonardo Mancini, Francesco Tarallo, Vincenzo Quinzi, Adriano Fratini, Stefano Mummolo, Enrico Marchetti

**Affiliations:** Department of Life, Health and Environmental Sciences, University of L’Aquila, 67100 L’Aquila, Italy; Francesco.tarallo93@gmail.com (F.T.); Vincenzo.quinzi@univaq.it (V.Q.); Adriano.fratini@graduate.univaq.it (A.F.); Stefano.mummolo@univaq.it (S.M.); enrico.marchetti@univaq.it (E.M.)

**Keywords:** root coverage, gingival recession, PRF, platelet-rich fibrin, coronally advanced flap, connective tissue graft

## Abstract

*Background and Objectives*: The aim of the present systematic review and meta-analysis was to investigate the efficacy of leukocyte–platelet-rich fibrin (L-PRF) in addition to coronally advanced flap (CAF) for the treatment of both single and multiple gingival recessions (GRs) compared to the CAF alone and to the adjunct of connective tissue graft (CTG). Root coverage outcomes using platelet concentrates have gained increased interest. In particular, it has been suggested that adding L-PRF to CAF may provide further benefits in the treatment of GRs. *Materials and Methods*: An electronic and manual literature search was conducted to identify randomized controlled trials (RTCs) investigating root coverage outcomes with CAF + L-PRF. The outcomes of interest included mean root coverage (mRC), recession reduction, keratinized tissue width (KTW) gain, gingival thickness (GT) gain, and patient-reported outcome measures (PROms) such as pain perception and discomfort. *Results*: A total of 275 patients and 611 surgical sites were analyzed. L-PRF in adjunct to single CAF seems to show statistically significant results regarding clinical attachment level (CAL) with a weighted means (WM) 0.43 95% CI (−0.04, 0.91), *p* < 0.0001, GT (WM 0.17 95% CI (−0.02, 0.36), *p* < 0.0001, and mRC (WM 13.95 95% CI (−1.99, 29.88) *p* < 0.0001, compared to single CAF alone. Interesting results were obtained from the adjunct of PRF to multiple CAF with respect to multiple CAF alone with an increase in the mRC WM 0.07 95% CI (−30.22, 30.35), *p* = 0.0001, and PPD change WM 0.26 95% CI (−0.06, 0.58), *p* < 00001. On the other hand, no statistically significant data were obtained when L-PRF was added to single or multiple CAF combined with CTG according to the included outcomes such as mRC (*p* = 0.03 overall). *Conclusions*: L-PRF is a valid alternative to CAF alone. L-PRF compared to CTG in single and multiple CAF showed statistically significant results regarding pain perception and discomfort PROms (*p* < 0.0001). However, CTG remains the gold standard for treating gingival recession.

## 1. Introduction

Gingival recession (GR) has been defined as the apical shift of the gingival margin in respect to the cementoenamel junction with concomitant exposure of the root surface in the oral cavity [1]. Several etiological factors such as tissue phenotype, gingival thickness, brushing technique, non-carious and carious cervical lesions, and periodontal predisposition were identified for this condition [2], which may account for its relatively high incidence in the population (45%) [3,4,5]. Root coverage procedures have been shown to be effective in treating single and multiple GRs [6,7,8], with large evidence supporting the superiority of coronally advanced flap (CAF) combined with connective tissue graft (CTG) [9,10,11]. Indeed, CAF + CTG provides the best mean root coverage (mRC) and aesthetic results, compared to alternative procedures such as free gingival graft or tunnel techniques [7,12], with clinical outcomes stable also in the long term [7,12]. Nevertheless, patient morbidity, the need for a second surgical site, and its limited availability are the main drawbacks that have been largely described for CTG [13,14].

Therefore, it is not surprising that a large variety of CTG substitutes were explored, including acellular dermal matrix, xenogeneic collagen matrix, and living cellular constructs. [15,16,17,18]. Moreover, although these materials showed superior patient-related outcomes (pain perception and discomfort) than CTG-based treatments, their clinical results were still inferior to the autogenous graft [6,16,19].

It has been advocated that the biologic agents may improve both soft tissue healing and the outcomes of the CAF [19,20]. Among them, platelets concentrates have gained increased interest. The first generations of platelets concentrates as platelet-rich plasma (PRP) due to the handling and the scarce adaptability to the root coverage procedure were not so popular among clinicians. Indeed, nowadays it is used more for aesthetic problems and in the regeneration of the skin. However, leukocyte–platelet-rich fibrin (L-PRF), or Choukroun’s PRF, represents the second generation of platelet concentrates and they are obtained from the centrifugation of blood (2700/3000 rpm for 12 min) without the addition of anticoagulants according to Choukroun et al. [21]. It has been shown that PRF can release growth factors such as platelet-derived growth factor (PDGF), vascular endothelial growth factor (VEGF), and insulin-like growth factor (I-LGF) that can promote better wound healing [20,21]. For the treatment of GRs, PRF has been used alone [22,23] or in combination with CTG [24]. Nowadays, several alternative protocols are suggested such as the horizontal centrifugation or the adding, inside the fibrin cloth, of substances such as albumin or titanium particles to increase the resorption time and the release of growth factors [25,26]. Nevertheless, this review aims to investigate and analyze the effect of the most diffused and popular second generation of platelet concentrates as L-PRF in single and multiple coronally advanced flap. The second outcome is the comparison with CTG which, as expressed before, is still a gold standard in root coverage.

## 2. Materials and Methods

### 2.1. Protocol Registration and Reporting

The present systematic review was conducted according to the guidelines of the Cochrane Handbook for Systematic Reviews of Interventions [27] and reported with the PRISMA guidelines [28,29]. The method of the analysis and inclusion criteria were specified in advance and registered on the Review Registry^®^ Identifying Number (916) [30].

### 2.2. Objectives

The goal of this systematic review was to evaluate the effects of L-PRF on root coverage outcomes in both single and multiple recessions.

### 2.3. Population, Intervention, Comparison, Outcome, Time (PICOT) Question

The following PICOT scheme was used to guide the inclusion and exclusion of the studies for the questions mentioned before [31].

Population (P): Patients presenting with single/isolated gingival recessions or with multiple adjacent gingival recessions (MAGRs) classified as recession type I [1,32].

Intervention (I): Root coverage procedures using L-PRF in combination with CAF.

Comparison (C): CAF alone vs. CAF + L-PRF or CAF + L-PRF vs. CAF + CTG in single and multiple recession defects.

Outcome (O): Mean root coverage (mRC), recession reduction (Rec Red), keratinized tissue width (KTW) gain, gingival thickness (GT) gain, as primary, and probing pocket depth (PPD) change, clinical attachment level (CAL) gain, patient-reported outcome measures (PROms) as secondary.

Time (T): Studies with a follow-up period of minimum six months.

### 2.4. Eligibility Criteria

Only randomized clinical trials (RCTs) with defined protocol were included in the present study. A list of inclusion and exclusion criteria was set to observe and assess significant heterogeneity in the selection of trials according to their design, selection criteria, interventions, and postsurgical treatment.

#### 2.4.1. Inclusion Criteria

- RCTs
- Treatment of single or multiple gingival recessions type I using CAF + L-PRF with a control group (CAF alone or CAF + CTG)
- Prospective interventional human studies
- Evaluation and reporting minimum primary clinical outcomes of interest (mRC, KTW gain, GT gain) over a minimum follow-up period of six months

#### 2.4.2. Exclusion Criteria

- Non-randomized studies (non-randomized prospective studies, case control studies, case series, case reports, previous systematic reviews)
- Studies using flap designs other than CAF
- Studies with less than six months of follow-up
- Studies that provided no data for the outcomes of interest
- Studies with a sample size of less than five patients per treatment arm
- Studies with unequal treatment between test and control (e.g., different sutures, different incision, periodontal dressing just on a site)

### 2.5. Information Sources and Search

A detailed computerized search was conducted on various databases (PubMed, Scopus, Cochrane, Clinicaltrials.gov) to identify eligible RCTs, followed by additional manual searching in relevant journals. Free terms, MeSH terms, and keywords related to: “platelet-rich fibrin”, “PRF”, “L-PRF”, “Miller Class I”, “coronally advanced flap”, “Miller Class II”, “gingival recession”, and “root coverage” were used to start the screening.

No restrictions were assigned regarding the date of publication, journal, or the language used. The search results were downloaded to a bibliographic database to facilitate duplicate removal and cross-reference checks. The last search was conducted on Medline 2 March 2020.

The electronic search was completed by an additional manual search of the following journals: Journal of Clinical Periodontology, Journal of Periodontology, Journal of Periodontal Research, Clinical Oral Investigations, and International Journal of Periodontics and Restorative Dentistry. The manual search in the referenced sources was performed from 1 January 2019 to 2 March 2020. Additionally, reference lists of the retrieved studies for full-text screening and previous reviews investigating periodontal plastic procedures were also screened.

### 2.6. Study Selection

Eligibility was assessed by two precalibrated review authors (LM and FT); initially title and abstract of the articles were screened. A full read of the remaining studies was performed to assess their alignment with the predetermined inclusion criteria. The screening and the assessment of eligibility of the studies were appraised through the use of Covidence (Covidence systematic review software, Veritas Health Innovation, Melbourne, Australia) [33].

### 2.7. Data Extraction and Management

Two authors (FT and LM) independently extracted data (authors, year of publication, study design, sample size, sample composition by sex and age, presence of control group, method of assessment, follow-up period, inclusion and exclusion criteria) from the selected studies using a predetermined extraction form [34]. At each stage, any debate between the reviewers was resolved through discussion and consensus. If a disagreement persisted, the judgment of a third reviewer (EM) was decisive. Aside from the primary outcomes (mRC, KTW gain, GT gain), the following study details were extracted:

- Type of study, number of centers, geographic location, sample frame (university vs. private practice), source of funding
- Population characteristics, age of participants, number of participants and treated sites (baseline/follow-up), singular/multiple treated sites, duration of follow-up
- Type of intervention, presurgical procedures, utilization of a graft material, and the type of graft
- PPD change, CAL gain, and PROms (using a visual analogue scale for pain perception and discomfort)

Means and standard deviations were extracted from the included RCTs.

### 2.8. Data Synthesis

The extracted data were subjected to a qualitative and quantitative analysis. The qualitative analysis and all the population-related data were recollected. The data for the quantitative assessment were extracted for each primary and secondary outcome, if present, and subjected to metanalysis. A confidential interval (CI) 95% was assessed and weighted means (WM) were used to synthesize the data according to mean root coverage (mRC), recession reduction (Rec Red), keratinized tissue width (KTW) gain, gingival thickness (GT) gain, probing pocket depth (PPD) change, clinical attachment level (CAL) gain, and patient-reported outcome measures (PROms).

### 2.9. Risk of Bias in Individual Studies

All included studies were evaluated according to the quality and risk of bias assessment tool (QAI) of Cochrane collaboration group [35]. QAI was based on seven stringent criteria. A scoring system was incorporated to assess an objective quality. Each study earned one point if the answer to the corresponding criterion was positive, none if the answer was negative or unclear. A study was considered at “low risk of bias” when random allocation, defined inclusion/exclusion criteria, blinding to patient and examiner, balanced experimental groups, identical treatment between groups (except for the intervention), and reporting of follow-up were present. Studies that met six criteria were considered to have a potentially moderate risk of bias. If two or more of these seven criteria were absent, the study was regarded to have a high risk of bias.

### 2.10. Risk of Bias across Studies

The heterogeneity of the studies was assessed with the use of the Cochran Q test, and for the proportion of inconsistency in the combined estimates due to between-study heterogeneity an I2 test was used. I2 values lower than 30% were representing low heterogeneity, values of 30% up to 60% as moderate heterogeneity, and values over 60% as substantial heterogeneity. Publication bias was assessed through the visualization of asymmetry on a funnel plot.

## 3. Results

### 3.1. Study Selection

**Figure 1:**
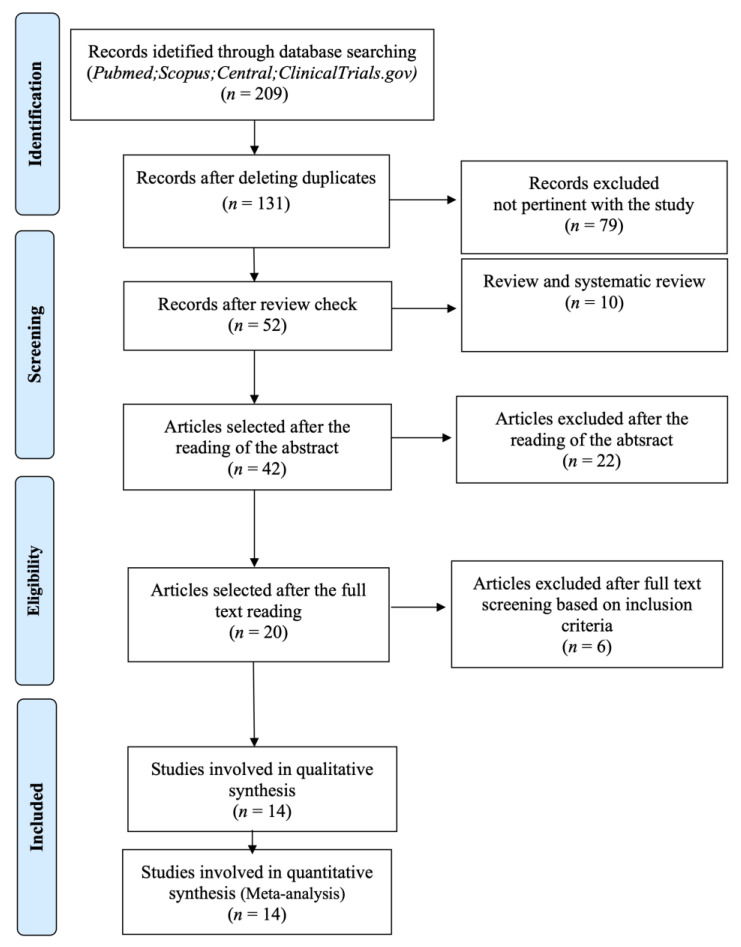
PRISMA flowchart for study selection.

Figure 1 shows the search process for selection of the included studies. The initial search provided a total of 209 articles following duplicates removal. Then, 131 articles were screened on the basis of titles and abstracts. Full-text assessment was performed on 20 articles. Among them, 6 studies [36,37,38,39,40,41] were excluded, due to their study design [36], surgical technique (e.g., orthodontic button for the suturing phase or microsurgical approach only for the test group) [37,38,39], use of concentrated growth factor instead of L-PRF [40], or data not reported [41]. Therefore, 14 trials [42,43,44,45,46,47,48,49,50,51,52,53,54,55] were included in the present systematic review.

### 3.2. Assessment of the Risk of Bias across Studies

**Figure 2:**
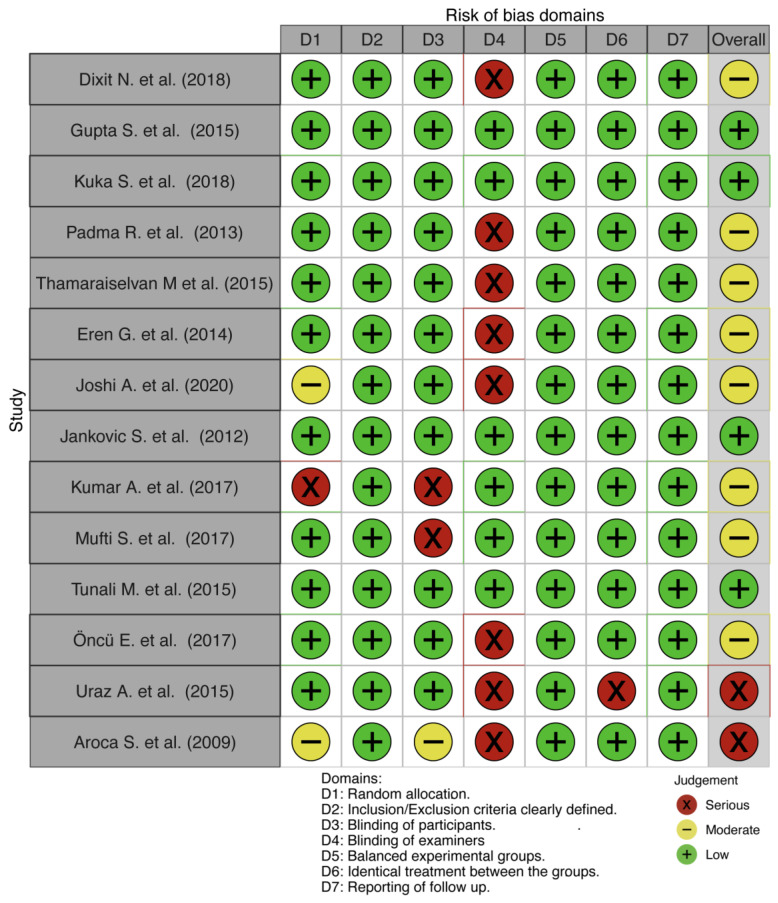
Assessment of the risk of bias.

Four studies were considered low risk of bias [42,43,44,45], eight [46,47,48,49,50,51,52,53] as moderate, and two [54,55] as high risk of bias (Figure 2). The studies were assessed based on random sequence generation, inclusion/exclusion criteria clearly defined, blinding of participants, blinding of examiners, balanced experimental groups, identical treatment between the groups, and follow up. These domains were graded as high, unclear, or low risk based on individual assessments.

**Figure 3:**
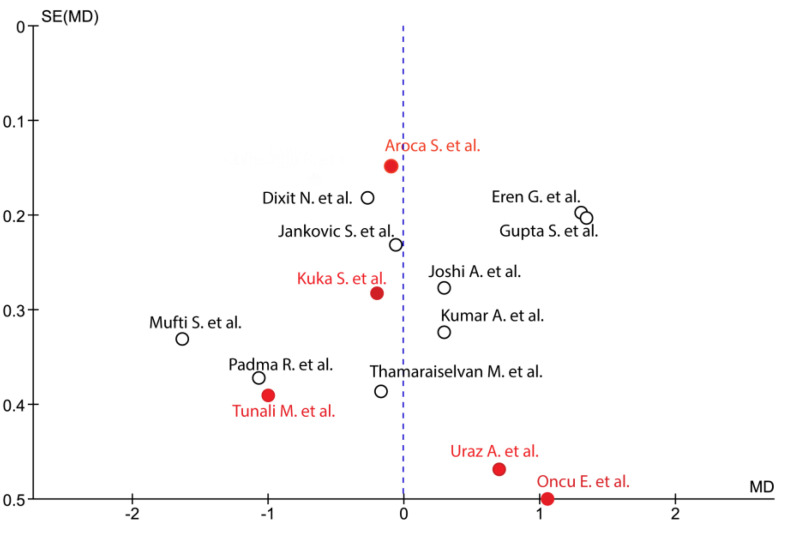
Funnel plot showed heterogeneity among the studies according to the standard error (SE) and the mean difference (MD). Black circle: single recession; red circle: multiple recession.

Heterogeneity was moderate or large for most of the comparisons. Visual inspection of the funnel plot revealed a certain degree of asymmetry. Studies with null or negative effects were also included (Figure 3). The heterogeneity grade was assessed with I^2^: low risk with I^2^ under 30%, moderate between 30% and 60%, and substantial heterogeneity with values of over 60%.

### 3.3. Study Characteristics

Table 1 depicts the characteristics of the included studies. Nine trials had a split-mouth design [43,45,46,47,48,51,52,54,55], while the remaining five had a parallel-arms design [42,44,49,50,53].

Nine studies treated isolated gingival recessions [46,47,48,49,50,52,53], while the others multiple adjacent gingival recessions (MAGRs) [42,43,51,54,55]. For the treatment of isolated gingival recessions, five articles compared the effect of L-PRF to CAF alone [44,46,48,52,53], while the other studies investigated the outcomes of CAF + L-PRF vs. CAF + CTG [45,47,50]. The study of Kumar et al. 2017 was designed as a three-arm trial, comparing CAF + L-PRF, CAF + CTG, and CAF alone [49].

Five studies evaluated the efficacy of L-PRF in MAGRs [42,43,51,54,55]. Three of them compared CAF + L-PRF vs. CAF + CTG [43,51,54], while two studies used CAF + L-PRF and CAF alone as test and control groups, respectively [42,55].

Four studies used periodontal dressing to promote undisturbed wound healing [43,44,45,49]. A similar postoperative regimen was observed in the included studies, with antibiotics, painkillers, and chlorhexidine digluconate prescribed for the first two postsurgical weeks. However, different dosage (amoxicillin 500 or 1000 mg) and painkiller medications (naproxen 550 mg or ibuprofen 400 mg) were reported. Five studies reported a mechanical handling of the L-PRF membranes [42,47,50,51,54] with the use of a PRF box, four studies a manual handling with woven gauze [43,44,46,48], and five did not specify the membrane handling [45,49,52,53,55].

Outcomes of interest, follow-up time points, and the centrifugation speed for L-PRF preparation are described in Table 2. The centrifugation speed was either 2700 or 3000 revolutions per minute (rpm) for 10 or 12 min. Five trials reported the spin protocol according to Choukroun et al. 2006 [21], and nine according to Pinto et al. 2019 [56]. Three trials reported data on patient discomfort following the surgical procedure using a visual analogue scale (VAS) [45,48,49,50]. Thirteen studies [42,44,45,46,47,48,49,50,51,52,53,54,55] had a maximum follow-up of 6 months, while only one article [43] had a 12-month follow-up.

### 3.4. Qualitative Analysis

When the outcomes of CAF + L-PRF were assessed in comparison with CAF alone for single gingival recessions, a greater but not statistically significant high mRC favoring the L-PRF group was found in two studies [44,53]. Padma et al. reported a statistically significant gain in mRC for CAF + L-PRF than CAF alone [52]. Significant KTW increase favoring CAF + L-PRF was shown in one study [52], while two trials found also a significantly greater GT gain in sites that received CAF + L-PRF compared to CAF alone [46,53]. Kumar et al. (2017) observed increased healing scores in sites treated with PRF [49].

Trials comparing CAF + L-PRF to CAF + CTG for single gingival recessions found higher mRC for the CTG group [45,48]. CTG-treated sites also showed greater KTW gain and GT gain [45,47,48,49,50]. Jankovic et al. 2012 reported better healing indexes and less discomfort for the L-PRF group compared to the CTG group [45]. Mufti et al. 2017 found greater healing scores for L-PRF-treated sites at 1 and 2 weeks [50]. Kumar et al. 2017 found better patient-reported esthetic scores and comfort for CAF + L-PRF compared to CAF + CTG [49].

Kuka et al. 2018 investigated the root coverage outcomes of MAGRs treated with CAF + L-PRF vs. CAF alone, showing a statistically significant gain in GT (0.53 ± 0.05 mm vs. 0.07 ± 0.05 mm) and marginally significant benefits in mRC (88.36 ± 15.45 % vs. 74.63 ± 8.05 %) for L-PRF-treated sites compared to sites that received CAF alone, respectively [23]. No significant differences between the two arms for PD change, KTW gain, CAL gain, and complete defect coverage were found. CAF + PRF showed a higher mRC than CAF alone (7.80 ± 1.32 vs. 7 ± 0, respectively), although this difference was not statistically significant [42].

One trial investigating MAGRs treated with CAF + L-PRF or CAF + CTG showed higher mRC and KTW gain for CAF + CTG [51] and a significant increase in GT in sites treated with L-PRF [51].

### 3.5. Root Coverage Outcomes of Single Gingival Recessions: CAF + L-PRF vs. CAF Alone

A borderline significant trend towards a higher mRC favoring CAF + L-PRF vs. CAF alone was observed, with an inverse variance-weighted means (WM) of 13.95 (95% CI [−1.99 to 29.88], *p* = 0.09).

**Figure 4:**
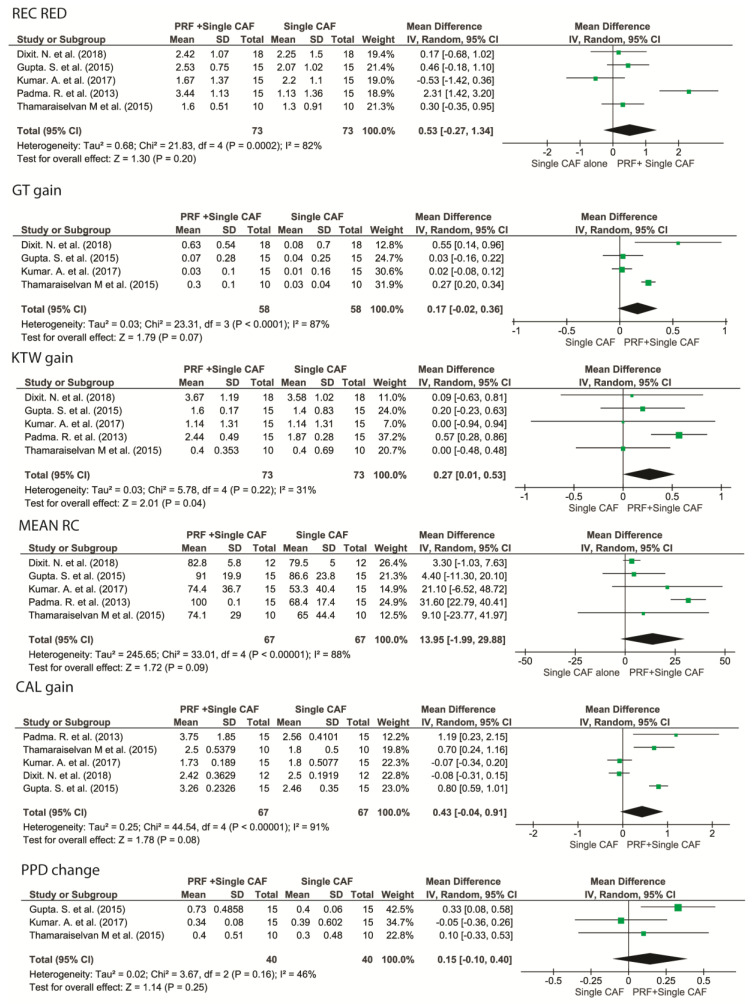
Forest plots comparing the effects of the adjunct of L-PRF to a single CAF versus single CAF alone. Mean RC, mean root coverage; REC RED, recession reduction; CAL gain, clinical attachment level gain; GT gain, gingival thickness gain; KTW gain, keratinized tissue width gain; PPD change, periodontal probing depth change.

No statistically significant differences were found between CAF + L-PRF vs. CAF alone in terms of Rec Red, PPD change, and KTW gain. CAF + L-PRF showed a mildly significantly higher GT gain compared to CAF alone, with a WM of 0.17 (95% CI [−0.02 to 0.36], *p* = 0.07). The addition of L-PRF also resulted in a statistically significantly greater CAL compared to flap alone (WM 0.52, 95% CI [0.13 to 0.91], *p* < 0.01) (Figure 4).

#### 3.5.1. Root Coverage Outcomes of Single Gingival Recessions: CAF + L-PRF vs. CAF + CTG

**Figure 5:**
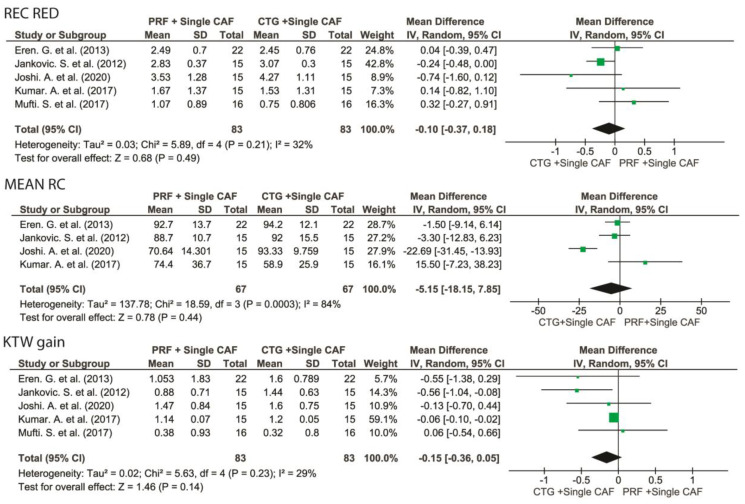
Forest plots of primary outcomes comparing the effects of the adjunct of L-PRF to a single CAF versus CTG + single CAF alone. Mean RC, mean root coverage; REC RED, recession reduction; KTW gain, keratinized tissue width gain.

No statistically significant differences were found between CAF + L-PRF vs. CAF + CTG alone in terms of mRC, Rec Red; CAF + CTG showed a marginally significantly greater KTW gain compared to CAF + L-PRF, with a WM of −0.15 mm (95% CI [−0.36 to 0.05], *p* = 0.07) (Figure 5).

#### 3.5.2. Root Coverage Outcomes of Multiple Adjacent Gingival Recessions: CAF + L-PRF vs. CAF Alone

No statistically significant differences were observed between CAF + PRF and CAF alone for the treatment of MAGRs in terms of mRC, KTW gain, and PPD change.

**Figure 6:**
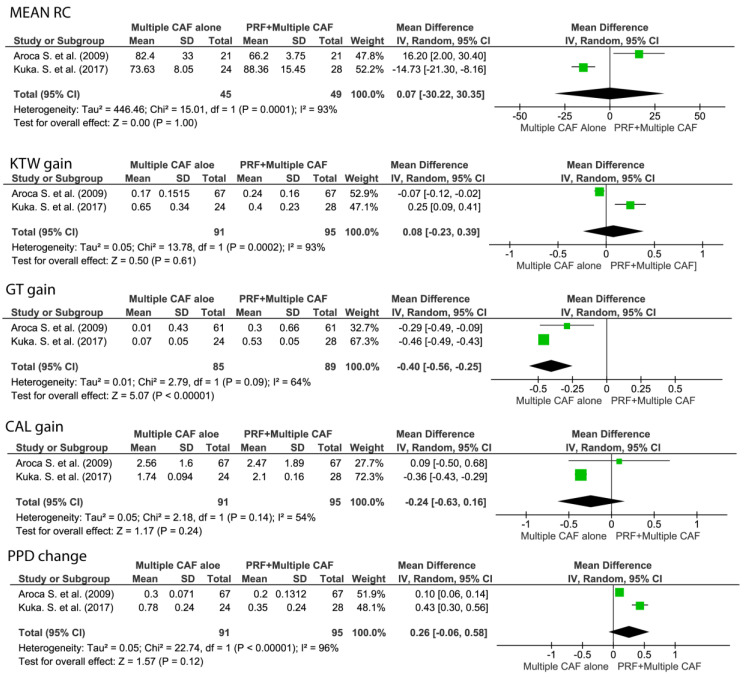
Forest plots for five parameters regarding the comparison between multiple CAF + L-PRF versus multiple CAF alone. Mean RC, mean root coverage; CAL gain, clinical attachment level gain; GT gain, gingival thickness gain; KTW gain, keratinized tissue width gain; PPD change, periodontal probing depth change.

CAF + L-PRF achieved highly significantly greater GT gain (WM −0.46, 95% CI [−0.49 to −0.43], *p* < 0.001) and CAL gain (WM −0.34, 95% CI [−0.58 to −0.1], *p* < 0.01) than CAF alone as reported in Figure 6.

#### 3.5.3. Root Coverage Outcomes of Multiple Adjacent Gingival Recessions: CAF + L-PRF vs. CAF + CTG

**Figure 7:**
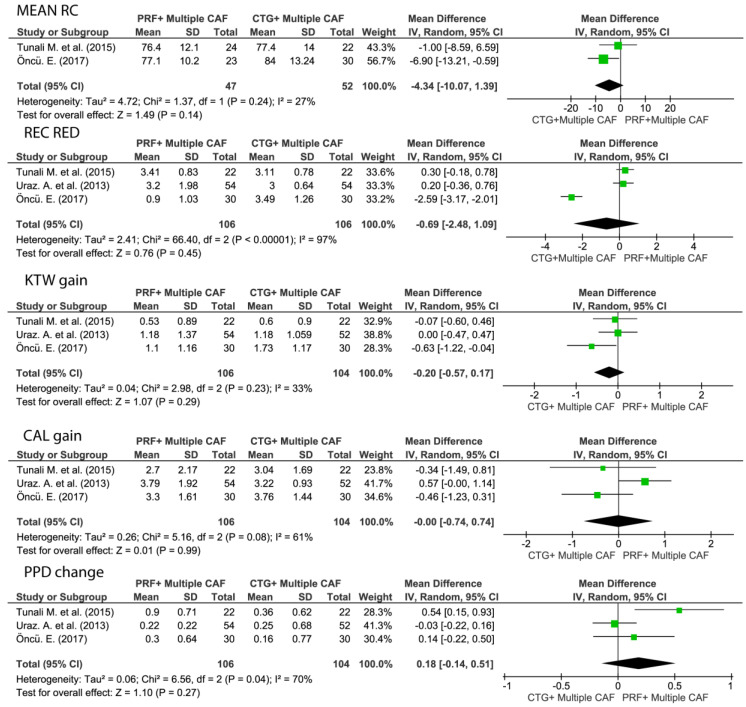
Forest plots for the clinical outcomes regarding the comparison between multiple CAF + L-PRF versus multiple CAF + CTG. Mean RC, mean root coverage; REC RED, recession reduction; CAL gain, clinical attachment level gain; KTW gain, keratinized tissue width gain; PPD change, periodontal probing depth change.

CAF + CTG exhibited statistically significantly superior mRC than CAF + L-PRF (WM −5.86, 95% CI [−11 to −0.73], *p* < 0.05) No statistically significant differences were found between the two groups in terms of Rec Red, KTW gain, CAL gain, and PPD change (Figure 7).

#### 3.5.4. Patient-Reported Postoperative Discomfort

**Figure 8:**
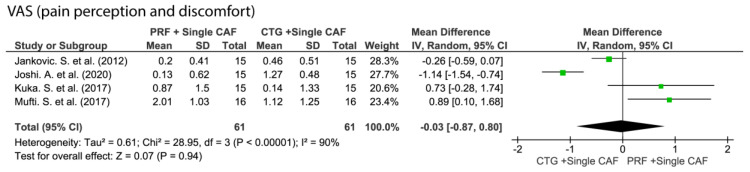
Forest plots for the VAS scale showing statistical difference between L-PRF and CTG in both single and multiple CAF technique.

Differences were found between CAF + L-PRF and CAF + CTG in terms of patient-reported morbidity (Figure 8). According to the PROms outcome, a VAS scale was used in these studies to assess the pain perception and discomfort. The use of L-PRF showed statistically significant data with respect to CTG with less pain and discomfort for the patients.

## 4. Discussion

Although it has been well demonstrated that CAF + CTG is the gold standard treatment for root coverage procedures [6,7,9], patient morbidity, the need for a second surgical site, and limited availability are the main drawbacks of autogenous grafts [13,14,16]. Therefore, it is not surprising that several soft-tissue-graft substitutes and biologic agents have been explored in the last two decades for the treatment of gingival recessions [18,20,57,58]. Among them, platelet concentrates have progressively gained popularity among clinicians due to their properties of enhancing wound healing [19,21]. While platelet-rich plasma and plasma rich in growth factor did not show promising root coverage outcomes [59], it has been advocated that the second generation of platelet concentrates L-PRF, involving the centrifugation of the blood without the addition of anticoagulants, can promote a greater release of growth factors, including platelet-derived growth factor, vascular endothelial growth factor, and transforming growth factor beta 1 [21,56]. Efficacy of platelet concentrates in promoting wound healing as in the treatment of osteonecrosis of the jaws is evident [60], and regarding tissue regeneration, is at the center of a recent academic debate [61]. Nevertheless, the effects of PRF in root coverage outcomes are still controversial [19].

### 4.1. Principal Findings

Results from our systematic review showed that PRF may provide superior mRC, KTW gain, GT gain, and healing scores compared to CAF alone. However, the meta-analysis confirmed statistically significantly better results for CAF + L-PRF over CAF alone for GT gain and CAL gain and mRC only. Due to its composition, with cells and growth factors, it has been speculated that L-PRF acts as a living cellular graft [21,56,62] and this may explain the improved outcomes compared to CAF alone.

Nevertheless, when PRF was compared to CTG for single gingival recessions, the findings from the systematic review were supporting the superiority of CTG in terms of mRC, KTW gain, and GT gain, although only the latter was significantly higher in the meta-analysis. Among its properties, CTG acts as a scaffold promoting the stabilization of the blood clot and increasing soft tissue thickness [10,63], which has been shown to be related to the amount of root coverage and its stability over time [12,64]. In addition, promoting the keratinization of the overlying epithelial layer is considered to be a prerogative of CTG only [10,65], which can explain the superior KTW gain commonly observed for the autogenous graft. The reduced thickness and stability of L-PRF compared to CTG may limit the soft tissue volume gain that can be achieved with the platelet concentrate. Moreover, in this study only RCTs with the application of a single layer of L-PRF were included.

The importance of KTW and GT gained following root coverage procedures has been demonstrated [6,12,17,66]. According to a recent 12-year follow-up from an RCT by Tavelli and coworkers, GT ≥ 1.2 mm at the six-month follow-up is a predictor for the stability of the gingival margin over time [17]. Interestingly, Barootchi et al. 2020 demonstrated that the augmented gingival thickness is sustained over time and is associated with a reduction in plaque index scores [18]. Therefore, although CTG remains the gold standard for increasing GT [18], we found that L-PRF resulted in significantly greater GT gain than CAF alone. It can be advocated that the addition of L-PRF can modify the periodontal soft tissue phenotype.

Interestingly, better healing scores, patient comfort, and reduced pain were observed for L-PRF compared to CTG. This can be due to the fact that CTG requires a second surgical site for the harvesting, with the palatal wound that can also have complications during the healing, including flap dehiscence, necrosis, bleeding, or excessive discomfort [13,14,63]. The release of growth factor promoted by the use of L-PRF and the accelerated wound healing may have also contributed to this result [21,56,62].

While the study of Kuka et al. 2018 showed superior mRC for MAGRs treated with CAF + L-PRF compared with CAF alone [42], Aroca et al. found significantly greater mRC for sites that did not receive the L-PRF (91.5% vs. 80.7%, respectively) [55]. However, when interpreting these results, it has to be considered that several factors have been shown to play a role in the root coverage outcomes of CAF, including tooth location, the amount of KTW and GT at baseline, and papillae dimensions [9,10,67]. Two studies included in the meta-analysis did not find significant differences in the root coverage outcomes of CAF + L-PRF and CAF + CTG for MAGRs [43,54], while another trial showed superior mRC and KTW gain for CTG-treated sites [51]. Therefore, definitive conclusions regarding the efficacy of L-PRF compared to CAF alone and CAF + CTG for the treatment of MAGRs cannot be drawn at the present time. It can be advocated that the addition of PRF may improve mRC and GT, even though it appears that CTG provides superior outcomes. On the other hand, our results demonstrated a statistically significantly lower morbidity for sites treated with PRF compared to CTG, both in single and multiple gingival recessions.

### 4.2. Agreement and Disagreement with Previous Reviews

Previous reviews analyzed the effect of L-PRF in comparison to single or multiple CAF focusing on clinical parameters and concluded that CTG provides the highest clinical and esthetic outcomes [7,8,11,18]. In line with the literature, we observed overall higher mRC, KTW gain, and GT gain for CAF + CTG, even though these findings did not reach a statistically significant difference in some of the included studies.

Moraschini et al. published a systematic review and meta-analysis in 2015 on the efficacy of PRF for the treatment of gingival recessions, based on six RCTs [68]. The reduced number of included studies was also a limitation of the review of Castro et al. 2017, Li et al., and Rodas et al. 2020 [69,70,71]. In addition, the reviews and meta-analyses available in the literature [68,69,70,71,72] include single and multiple sites in the same analysis, while it has been suggested to evaluate single and multiple gingival recessions separately in pairwise meta-analyses [73]. The latest systematic review according to Panda et al. 2020 analyzed only the effect of L-PRF to CAF and not also between L-PRF and CTG which is the standard in root coverage procedures. Moreover, in the meta-analyses, the inclusion of Dixit et al. 2018 [46] in the multiple recession group might affect the results for its design as a study for single and not multiple recessions [74].

Another possible drawback of previous reviews includes analyzing different platelet concentrates, such as platelet-rich plasma and concentrated growth factor (CGF), together with the PRF. As highlighted by Dogan et al. [34], while concentrated growth factor is obtained from the centrifugation of venous blood with platelets contained in a gel layer with fibrin matrix similarly to L-PRF, the different centrifugation speed for CGF allows for a higher amount of growth factor compared to L-PRF.

Miron et al. 2020 demonstrated that CAF + PRF achieved a statistically superior mRC than CAF alone, while the amount of root coverage was significantly lower when compared to CAF + CTG [72]. Similarly, CAF + CTG obtained a greater KTW gain than CAF + PRF [72]. These findings are in agreement with the results from our study. In addition, we demonstrated that CAF + PRF is significantly associated with lower patient morbidity than CAF + CTG.

### 4.3. Limitations and Recommendation for Future Research

Limitations of the present review include the number of trials that were considered as having moderate or high risk of bias, together with the moderate/large heterogeneity observed. Therefore, readers may take these aspects into consideration when interpreting our results. In addition, although 14 RCTs were included in the present analysis, the reduced number of articles with the same recession type (single or multiple) and control group (CAF alone or CAF + CTG) may have prevented finding statistically significant differences in the outcomes of interest. Only studies with 6- or 12-month follow-up are available in the literature when investigating the outcomes of PRF. Moreover, in this systematic review were included studies in which smoker patients were present and this might affect the regenerative procedure; another limitation is in the spin protocol and in the handling of the membrane which could be manual or mechanical. Recommendations for future research include increasing the number of high-quality RCTs assessing the efficacy of CAF + L-PRF compared with CAF alone or with CAF + CTG, studies following CONSORT guidelines for reducing the risk of bias, trials incorporating patient-reported outcome measures and long-term outcomes of sites treated with PRF. Mixed-modeling-based network meta-analyses evaluating the efficacy of L-PRF compared with all the other root coverage techniques described in the literature are encouraged.

## 5. Conclusions

Within its limitations, the present review suggests that L-PRF can provide additional benefits to CAF in terms of mRC and GT gain, while CAF + CTG showed superior mRC and KTW gain than CAF + L-PRF. Indeed, its use should be stimulated due to its potential biological benefits and reduced patient morbidity. Nevertheless, the limited number of studies in the pairwise comparisons may have prevented detecting other significant differences among the treatments. Future studies are therefore encouraged to further investigate the efficacy of L-PRF in root coverage procedure.

## Figures and Tables

**Table 1:** Characteristics of the included studies. PRF, platelet-rich fibrin; CAF, coronally advanced flap; CTG, connective tissue graft.

Authors (year)	Study Design	Mean Age (years)	n (M)	n (F)	n. of Patients	Surgical Sites	Study Group (s)	Control Group	Recession Type
Dixit N. et al. (2018) [46]	Split-mouth	18–50	-	-	12	36	L-PRF + CAF (18)	CAF alone (18)	Single
Gupta S. et al. (2015) [44]	Parallel	20–50	16	10	26	30	L-PRF + CAF (15)	CAF alone (15)	Single
Padma R. et al. (2013) [52]	Split-mouth	18–35	-	-	15	30	L-PRF + CAF (15)	CAF alone (15)	Single
Thamaraiselvan M et al. (2015) [53]	Parallel	21–47	18	2	20	20	L-PRF + CAF (10)	CAF alone (10)	Single
Eren G. and Atilla G. (2014) [47]	Split-mouth	18–52	9	13	22	44	L-PRF + CAF (22)	CTG + CAF (22)	Single
Joshi A. et al. (2020) [48]	Split-mouth	18–40	-	-	15	30	L-PRF + CAF (15)	CAF alone (15)	Single
Jankovic S. et al. (2012) [45]	Split-mouth	19–40	5	10	15	30	L-PRF + CAF (15)	CTG + CAF (15)	Single
Kumar A. et al. (2017) [49]	Parallel	-	34	2	36	45	L-PRF + CAF (15); CAF alone (15)	CTG + CAF (15)	Single
Mufti S. et al. (2017) [50]	Parallel	37.56 ± 5.29	9	7	16	32	L-PRF + CAF (16)	CTG + CAF (16)	Single
Tunali M. et al. (2015) [43]	Split-mouth	25–52	4	6	10	44	L-PRF + CAF (22)	CTG + CAF (22)	Multiple
Öncü E. et al. (2017) [51]	Split-mouth	20–60	9	11	20	60	L-PRF + CAF (30)	CTG + CAF (30)	Multiple
Uraz A. et al. (2015) [54]	Split-mouth	23–48	9	6	20	106	L-PRF + CAF (54)	CTG + CAF (52)	Multiple
Aroca S. et al. (2009) [55]	Split-mouth	21–41	6	15	24	52	L-PRF + CAF (28)	CAF alone (24)	Multiple
Kuka S. et al. (2018) [42]	Parallel	21–41	11	13	24	52	L-PRF + CAF (28)	CAF alone (24)	Multiple

**Table 2:** Characteristics of the included studies according to the clinical parameters and the follow-up period. CAF, coronally advanced flap; L-PRF, leukocyte–platelet-rich fibrin; CTG, connective tissue graft; mRC, mean root coverage; REC, recession; CAL, clinical attachment level; GT, gingival thickness; KTW, keratinized tissue width; PPD, periodontal probing depth; VAS, visual analogue scale.

**Single CAF + L-PRF vs. Single CAF Alone**				
**Authors (year)**	**Clinical Parameters**	**Recording Data Time**	**Presurgical Procedure**	**Centrifugation Speed**
Dixit N. et al. (2018) [46]	mRC; REC; CAL; GT; KTW	Baseline, 1 month, 3 months, 6 months	Scaling and root planing	2700 rpm for 12 min
Gupta S. et al. (2015) [44]	mRC; REC; CAL; GT; KTW; PPD	Baseline, 1 month, 3 months, 6 months	Scaling and root planing	2700 rpm for 12 min
Padma R. et al. (2013) [52]	mRC; REC; CAL; KTW	Baseline, 1 month, 3 months, 6 months	Scaling and root planing	3000 rpm for 10 min
Thamaraiselvan M et al. (2015) [53]	mRC; REC; CAL; GT; KTW	Baseline, 6 months	Scaling and root planing	3000 rpm for 10 min
Kumar A. et al. (2017) [49]	REC; mRC; CAL; GT; KTW; VAS; PPD	Baseline, 6 months	Scaling and root planing	3000 rpm for 12 min
**Single CAF + L-PRF vs. Single CAF + CTG**				
**Authors (year)**	**Clinical Parameters**	**Recording Data Time**	**Presurgical Procedure**	**Centrifugation Speed**
Eren G. and Atilla G. (2014) [47]	REC; mRC; CAL; GT; KTW	Baseline, 6 months	Oral hygiene	3000 rpm for 12 min
Joshi A. et al. (2020) [48]	mRC; REC; CAL; GT; KTW; PPD	Baseline, 6 months	Scaling and root planing	3000 rpm for 10 min
Jankovic S. et al. (2012) [45]	REC; mRC; CAL; GT; KTW; PPD	Baseline, 6 months	Scaling and root planing	3000 rpm for 10 min
Kumar A. et al. (2017) [49]	REC; mRC; CAL; GT; KTW; VAS; PPD	Baseline, 6 months	Scaling and root planing	3000 rpm for 12 min
Mufti S. et al. (2017) [50]	REC; CAL; GT; KTW; VAS	Baseline, 6 months	Scaling and root planing	3000 rpm for 10 min
**Multiple CAF +L-PRF vs. Multiple CAF + CTG**				
**Authors (year)**	**Clinical Parameters**	**Recording Data Time**	**Presurgical Procedure**	**Centrifugation Speed**
Tunali M. et al. (2015) [43]	REC; mRC; CAL; KTW; PPD	Baseline, 6 months, 12 months	Scaling and root planing	2700 rpm for 12 min
Öncü E. et al. (2017) [51]	mRC; CAL; KTW; REC; PPD	Baseline, 6 months	Oral hygiene	2700 rpm for 12 min
Uraz A. et al. (2015) [54]	REC; mRC; CAL; KTW; PPD	Baseline, 6 months	Scaling and root planing	2700 rpm for 12 min
**Multiple CAF +PRF vs. Multiple CAF**				
**Authors (year)**	**Clinical Parameters**	**Recording Data Time**	**Presurgical Procedure**	**Centrifugation Speed**
Kuka S. et al. (2018) [42]	REC; mRC; CAL; GT; KTW; PPD	Baseline, 3 months, 6 months	Scaling and root planing	3000 rpm for 10 min
Aroca S. et al. (2009) [55]	REC; mRC; CAL; GT; KTW; PPD.	Baseline, 3 months, 6 months	Scaling and root planing	3000 rpm for 10 min

## Data Availability

The data presented in this study are openly available.
